# Notes and News

**Published:** 1905-07-22

**Authors:** 


### Notes and News.

The Metropolitan Hospital Sunday Fund collection now amounts to about £57,000.

The first of a number of day nurseries has been opened in Myrtle Street, Hoxton. Children are admitted from four weeks old, and are cared for and fed during the day, a small charge being made.

By the will of Mrs. Florence Mary Hames (wife of Mr. George Henry Hames, F.R.C.S.), King Edward's Hospital Fund for London will eventually receive, after certain bequests have been made, the residue of her estate, which, it is understood, will exceed £50,000.

From July 19th to 23rd, the Royal Institute of Public Health will hold a Congress at the King's College, and at the Polytechnic, Regent Street. The inaugural meeting will be presided over by Lord Londonderry, at His Majesty's Theatre, on the afternoon of the 19th, at 3 p.m., when the public will be admitted to the upper circle. All particulars may be obtained from the office of the Institute, Russell Square.

The committee of the Great Ormond Street Hospital for Sick Children announce that they have opened a ward for the exclusive use of children under two years suffering from ailments due to the hot weather. We hope this excellent arrangement will be widely known and taken advantage of. The lives of many young children could be saved if the mothers would take them promptly to the hospital for treatment, and district visitors and the clergy should assist in educating the parents to the necessity of so doing.

The French Ambassador last week unveiled at the French Hospital, Shaftesbury Avenue, a bust of the late Dr. Achille Vintras, the founder and senior physician of that institution. The bust, which is affixed to the wall on the left of the main entrance, has been executed and presented by M. Lanteri, the sculptor, who is a member of the hospital committee; underneath is the inscription, "Au Docteur Achille Vintras, Fondateur et Docteur en chef de l'Hôpital Français à Londres. La Colonie Française reconnaissante 1905."

The Chairman of the Executive Committee of King Edward's Hospital Fund announces that, on condition £18,000 is received towards the fund by the end of this month, Mr. H. W. Marshall has offered a gift of land worth £12,000. The sum of nearly £8,000 is still required, and contributions will be gratefully received, before July 31st, at the Bank of England, or 81 Cheapside, E.C.

The Governors of the National Society for the Employment of Epileptics held their twelfth annual meeting recently at Denison House, Westminster, the London offices of the Colony, which is at Chalfont St. Peter. The chair was taken by Mr. E. Montefiore Nichoffs. The opening of a new Home for special benefit of Hampshire cases, and of an Administrative House, and various other buildings was reported.

At the last meeting of the Council of the Royal College of Surgeons, the following professors and lecturers were elected—Hunterian professors: Warrington Haward, A. H. Cheatle, H. J. Paterson, and C. G. Seligmann. As Arris and Gale Lecturers: J. H. Watson and Sydney W. Curl. As Erasmus Wilson Lecturer: James Sherren. It was decided to admit the application of the Cairo Medical School to be recognised as a place of study for candidates for the diplomas of the two Royal Colleges, the Royal College of Physicians having signified consent also.

The University of Liverpool is receiving the most substantial and gratifying encouragement, and is rapidly advancing towards a complete organisation. On the occasion of the Degree Day on July 8, the new electro-technical laboratories were opened by Sir Joseph Swan. These buildings have cost £12,000, provided by the University, and the cost of equipment, amounting to £1,000, has been defrayed by the Lancashire County Council. The University has recently been enriched by the addition to the chemical laboratories of a department for physical chemistry, a building for an study of natural history, and numerous benefactions of a substantial amount have been received. Large annual grants are promised, and donations have been made by several of the local authorities.

The Lord Mayor presided last week at a meeting at the Mansion House, held to celebrate the 21st anniversary of the opening of the Mary Wardell Convalescent Home, Brockley Hill, Stanmore. He said that Miss Wardell was to be congratulated, for it was given to only a few to originate and carry out successfully a great work for the public benefit. The work of the home was real and useful. Sir Douglas Powell observed that the institution supplied a distinct want, which was felt by the rich and poor alike. Sir W. Broadbent, remarked that the need for the home was very great, in fact indispensable. The community owed a deep debt of gratitude to Miss Wardell for establishing and carrying on the work for so many years. On the motion of Dr. Burnett, seconded by Sir Hugh Beevor, a resolution was passed expressing the desirability of raising an endowment fund for the future satisfactory working of the home and putting it on a sound financial footing.

